# Application of Nudges to Design Clinical Decision Support Tools: Systematic Approach Guided by Implementation Science

**DOI:** 10.2196/73189

**Published:** 2025-09-25

**Authors:** Katy E Trinkley, Danielle Maestas Duran, Shelley Zhang, Meagan Bean, Larry A Allen, Russell E Glasgow, Amy G Huebschmann, Chen-Tan Lin, Jason N Mansoori, Anna M Maw, James Mitchell, Laura D Scherer, Daniel D Matlock

**Affiliations:** 1 Department of Family Medicine, School of Medicine, University of Colorado Anschutz Medical Campus, 1890 North Revere Court, Aurora, CO, 80045, United States, 1 3037246563; 2 UCHealth Colorado, Aurora, CO, United States; 3 Adult and Child Center for Outcomes Research and Delivery Science, School of Medicine, University of Colorado Anschutz Medical Campus, Aurora, CO, United States; 4 Department of Biomedical Informatics, School of Medicine, University of Colorado Anschutz Medical Campus, Aurora, CO, United States; 5 Department of Cardiology, School of Medicine, University of Colorado Anschutz Medical Campus, Aurora, CO, United States; 6 Department of General Internal Medicine, School of Medicine, University of Colorado Anschutz Medical Campus, Aurora, CO, United States; 7 Ludeman Family Center for Women’s Health Research, School of Medicine, University of Colorado Anschutz Medical Campus, Aurora, CO, United States; 8 Division of Pulmonary and Critical Care Medicine, Denver Health Medical Center, Denver, CO, United States; 9 Division of Hospital Medicine, School of Medicine, University of Colorado Anschutz Medical Campus, Aurora, CO, United States; 10 Division of Geriatrics, School of Medicine, University of Colorado Anschutz Medical Campus, Aurora, CO, United States; 11 VA Eastern Colorado Geriatric Research Education and Clinical Center, Aurora, CO, United States

**Keywords:** behavioral economics, clinical decision support tools, heart failure, implementation science, nudges

## Abstract

**Background:**

Clinical decision support (CDS) is one strategy to increase evidence-based practices by clinicians. Despite its potential, CDS tools produce mixed results and are often disliked by clinicians. Principles from behavioral economics such as “nudges” may improve the effectiveness and clinician satisfaction of CDS tools. This paper outlines a pragmatic approach grounded in implementation science to identify and prioritize how to incorporate different types of nudges into CDS tools.

**Objective:**

The purpose of this paper is to describe a systematic and pragmatic approach grounded in implementation science to identify and prioritize how best to incorporate different types of nudges into CDS tools. We provide a case example of how this systematic approach was applied to design a CDS tool to improve guideline-concordant prescribing of mineralocorticoid receptor antagonists for patients with heart failure and reduced ejection fraction.

**Methods:**

We applied the Messenger, Incentives, Norms, Defaults, Salience, Priming, Affect, Commitments, and Ego nudge framework and the Practical, Robust Implementation and Sustainability Model implementation science framework to systematically and pragmatically identify and prioritize different types of nudges for CDS tools. To illustrate how these frameworks can be applied in a real-life scenario, we use a case example of a CDS tool to improve guideline-concordant prescribing for patients with heart failure. We describe a process of how these frameworks can be used pragmatically by clinicians and informaticists or more technical CDS builders to apply nudge theory to CDS tools.

**Results:**

We defined four iterative steps guided by the Practical, Robust Implementation and Sustainability Model: (1) engage partners for user-centered design, (2) develop a shared understanding of the nudge types, (3) determine the overarching CDS format, and (4) brainstorm and prioritize nudge types to address each modifiable contextual issue. These steps are iterative and intended to be adapted to align with the local resources and needs of various clinical scenarios and settings. We provide illustrative examples of how this approach was applied to the case example, including who we engaged, details of nudge design decisions, and lessons learned.

**Conclusions:**

We present a pragmatic approach to guide the selection and prioritization of nudges, informed by implementation science. This approach can be used to comprehensively and systematically consider key issues when designing CDS to optimize clinician satisfaction, effectiveness, equity, and sustainability while minimizing the potential for unintended consequences. This approach can be adapted and generalized to other health settings and clinical situations, advancing the goals of learning health systems to expedite the translation of evidence into practice.

## Introduction

Uptake of evidence-based medicine into routine practice is slow and rare [1]; it takes an estimated 17 years for just 14% of effective practices to be translated [2]. Learning health systems and the field of implementation science aim to expedite the translation of evidence into practice [3] and have had some success [4-9]. One strategy increasingly used to expedite knowledge translation is the use of clinical decision support (CDS) tools [10-13]. CDS tools are often automated within the electronic health record (EHR) and are designed to encourage clinicians to follow more evidence-based practices. While CDS interventions have shown promise in improving the translation of evidence into routine practice settings [14-19], barriers to their widespread acceptance include the need to make them more user-friendly for clinicians. Unfortunately, CDS tools are not consistently designed well with clinician needs and preferences in mind, which has led to significant clinician dissatisfaction with CDS and the phenomenon of “alert fatigue” [20-22]. Thus, opportunities remain [23] to optimize the design and implementation of CDS.

One increasingly popular opportunity to optimize CDS is to use principles from behavioral economics to augment CDS [24,25]. Behavioral economics principles draw upon cognitive science and social psychology and can be used to develop more user-friendly interventions [25]. Some of the most widely used behavioral economics tools are “nudges,” which alter the manner or environment in which decisions are made, otherwise known as “choice architecture” [25,26]. Strategic use of nudges within CDS can guide clinicians toward making guideline-concordant decisions without restricting their choice [26]. There are multiple examples of nudges positively promoting behavior change across diverse settings and populations [27]. We have found many examples of nudges being used for CDS, including efforts to increase statin prescribing during primary care visits using active choice prompts and monthly peer comparison feedback; strategies implemented across pharmacies in 37 different states to increase recombinant zoster vaccine second dose rates, which prioritized compliance with organizational goals and incentives; and efforts to increase the adoption of a pulmonary embolism risk prediction tool in an emergency department of a large academic health care system [28-31].

There are many factors to consider when applying nudges to CDS. As is the case for CDS tools generally, nudge solutions should be aligned with the specific clinical situation and consider the context, such as associated workflows, available resources, and perspectives of the target audience [15,32,33]. Nudges also need to be designed carefully to ensure positive ethical behavior change, avoid unintended consequences [34], and consider maintenance of behavior change [35]. If not designed well, nudges could lead to unintended consequences or pose ethical concerns.

Across a variety of research and operational implementation projects, we have observed the need for guidance on how to select and apply the different types of nudges, particularly guidance that is systematic, comprehensive, and standardized to facilitate alignment with the clinical context [20,36-38]. Such guidance is needed to enable selection of nudges that are effective, sustainable, ethical, generalizable, and adaptable. Such a guiding framework can help developers create CDS with embedded nudges that are both locally relevant and scalable. Further, guidance is needed to strategically select from among the many types of nudges that could be applied, those that are best aligned with the specific CDS situation and that will best guide decision-making to maximize impact.

The purpose of this paper is to describe a systematic and pragmatic approach grounded in implementation science to identify and prioritize how best to incorporate different types of nudges into CDS tools. We provide a case example of how this systematic approach was applied to design a CDS tool to improve guideline-concordant prescribing of mineralocorticoid receptor antagonists for patients with heart failure and reduced ejection fraction.

## Methods

### Overview

To develop our pragmatic approach, we convened a multidisciplinary group representing clinicians (primary care, geriatrics, cardiology, critical care), psychologists; informaticists; implementation scientists; health services researchers; and experts in human-computer interaction, decision science, and behavioral economics. Members of the group were purposefully selected based on their relevant expertise. They were contacted electronically by emails and synchronous meetings to discuss the goals of the approach, framework selection, and processes. This group was tasked with iteratively developing a systematic approach to select nudge “types” and design nudge “forms” for clinician-facing CDS tools within an EHR. Here, we define nudge “types” based on the Messenger, Incentives, Norms, Defaults, Salience, Priming, Affect, Commitments, and Ego (MINDSPACE) categories (see framework selection, below) and “forms” as different ways the nudge types can be designed [39].

At the outset, we defined key characteristics and goals of this approach including that it needed to (1) be feasible and pragmatic; (2) be applicable across different types of CDS and clinical situations; (3) comprehensively consider the breadth of different types of nudges; (4) balance the potential to impact clinician behavior change with the risk of unintended consequences and resources and effort required to build the nudge and CDS; and (5) promote or foster equity, sustainability, and generalizability of the nudge and overarching CDS solution.

### Framework Selection

To facilitate comprehensive consideration of different types of nudges, we selected the MINDSPACE framework [39]. While other frameworks could be used, we selected MINDSPACE because it is commonly used [40] and is easy to use for diverse audiences, including those without extensive training in psychology or behavioral economics. MINDSPACE categorizes nudges into 9 types based on how they alter the choice architecture. The MINDSPACE nudge types [39] are described in Table 1 along with the theoretical logic behind the behavior the nudge seeks to address. Each nudge type can then be applied in different ways or forms for specific situations. For example, the nudge type of salience can take on the form of bolded text or a pop-up that causes an interruption. To understand the potential effectiveness of different nudge types and forms on changing behavior, we also used the nudge “ladder” [25]. The nudge ladder categorizes nudges on an effectiveness scale of 1-5 (1=strongest/most effective) in which default options are the strongest and simply providing information is the weakest or least likely to change behavior [25]. As described in Table 1, we applied the nudge ladder ratings to the 9 MINDSPACE nudge types.

**Table 1.** MINDSPACE definitions for nudge types and their “ladder” strength with notes on how the study team came to a shared understanding.

MINDSPACE nudge type	MINDSPACE definition (mechanisms)	Notes from team	Place on ladder (1=least effective, 5=most effective)	Examples in CDS setting
Messenger	We are heavily influenced by who communicates information	Messenger also includes differential impacts on behavior when information is communicated by experts (real or perceived) versus novices, for example, or by organizational leaders or people with authoritative power versus peers	2	Insert language or institutional branding to indicate when the alert is personalized to the clinician’s institution or recommended by specific clinic leadership versus provided by a third-party data vendor Present reference links to clinical guidelines and literature from public health agencies or other governing bodies prominently within the alert
Incentives	Our responses to incentives are shaped by predictable mental shortcuts, such as strongly avoiding losses	No amendment necessary	4	Disable the alert if the clinician reaches a goal threshold for a behavior over a certain timeframe Require clinicians to provide a reason if they choose to defer action. Display reasons in a readily visible area of the patient’s chart to increase accountability.
Norms	We are strongly influenced by what others do	Amend definition to include: we are strongly influenced by norms (what others do), *both real and perceived*	2	Display the average frequency of a behavior or a target level set for the clinician’s specific practice site Present recommended orders in an order set
Defaults	We “go with the flow” of pre-set options	No amendment necessary	5	When the clinician opens an order set, display particular medication or lab orders based on the patient’s current therapies or lab-based criteria Default lab monitoring orders with appropriate sequencing as a panel in the alert
Salience	Our attention is drawn to what is novel and seems relevant to us	Amend definition to include**:** anything that draws our attention, not necessarily because it is novel or relevant: Things that stand out or draw our attention are more likely to influence our behavior	2	Present abnormal lab values in red bolded text or larger font size Incorporate the patient’s name or photo into alert body Using the current alert, trigger just-in-time communications to clinicians about future lab monitoring
Priming	Our acts are often influenced by subconscious cues	No amendment necessary	4	Remove native medication warnings that may be firing before the custom alert to remove negative priming mechanism within EHR^b^
Affect	Our emotional associations can powerfully shape our actions	No amendment necessary	2	Add more options for deferring the alert’s re-appearance to minimize psychological reactance Include statistics or a clinical vignette in the alert that describes the patient’s potential disease outcomes due to poor medication management
Commitments	We seek to be consistent with our public promises and reciprocate acts	No amendment necessary	3	Implement patient-facing alert that allows patient to indicate whether they are interested in receiving information about new therapy
Ego	We act in ways that make us feel better about ourselves	No amendment necessary	2	In the alert, display the clinician’s percentile ranking relative to peers in performing the recommended behavior

a MINDSPACE: Messenger, Incentives, Norms, Defaults, Salience, Priming, Affect, Commitments, and Ego

b EHR: electronic health record.

While other implementation science frameworks could be used, we selected the Practical, Robust Implementation and Sustainability Model (PRISM) [41-43] because (1) it has been applied with user-centered design (UCD) and human-computer interaction principles to CDS [32], (2) it offers specific guidance on issues of sustainability and equity [41,42,44-46], (3) it includes the RE-AIM (Reach, Effectiveness, Adoption, Implementation, Maintenance) outcome measures, which can guide nudge design decisions and support pragmatic evaluation of nudges deployed, and (4) there are numerous tools and resources to assist diverse users with and without implementation science expertise through the process of applying the PRISM framework [41,42,47,48].

Use of a framework such as PRISM is key to systematically design nudges to align with the implementation context to optimize relevance, generalizability, sustainability, and equity [41,42]. An implementation science framework such as PRISM provides holistic guidance on how to assess and align complex multilevel contextual issues with the selection of nudge types and forms, along with consideration and measurement of a variety of implementation outcomes.

**Figure 1.**
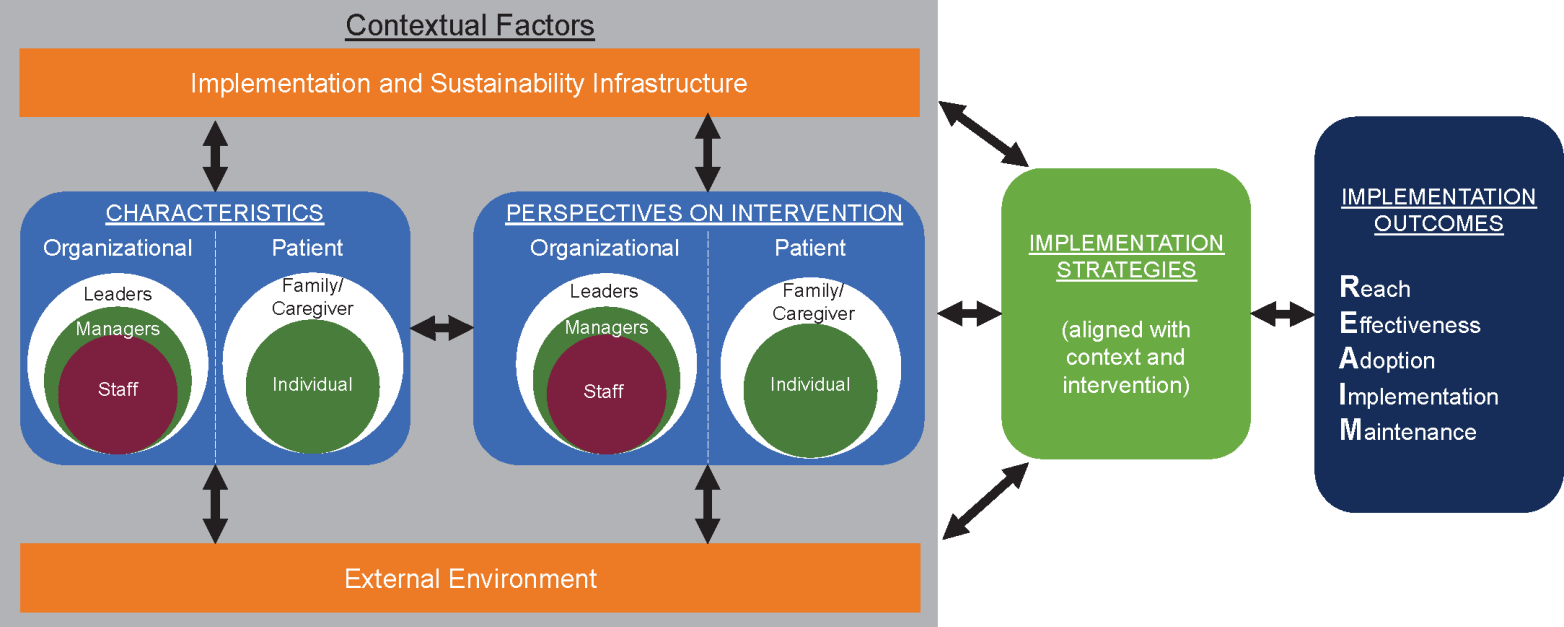
The PRISM implementation logic model. PRISM considers the dynamic interactions of the context at multiple levels of the organization, patient, or community along with factors outside the local setting (eg, external environment includes evidence-based guidelines, human-computer interaction best practices, principles of behavioral economics) and factors that influence the ability to implement or sustain the program. These contextual factors inform the design of implementation strategies (eg, fitting within usual workflows) and the intervention itself. In the case of CDS tools, the CDS can be the strategy or intervention depending on the framing of the project. Design decisions are made while proactively and iteratively considering the impact on PRISM’s RE-AIM outcomes, which include consideration of equity across all outcomes. Contextual factors and RE-AIM outcomes are informed by multilevel partner engagement across diverse perspectives to promote equity. CDS: clinical decision support; PRISM: Practical, Robust Implementation and Sustainability Model; RE-AIM: Reach, Effectiveness, Adoption, Implementation, Maintenance.

PRISM is illustrated in Figure 1 and includes the RE-AIM outcomes and 4 context domains that work together to enhance alignment among the context, intervention, and implementation strategies in order to maximize outcomes [43]. The PRISM context domains are (1) the characteristics of the organization and patient, (2) the perspectives of the organization and patient about the intervention including the complexity and evidence to support the intervention, (3) the implementation and sustainability infrastructure including resources available for initial and ongoing implementation, and (4) the external environment including clinical guidelines and policies or regulations.

Central to PRISM is consideration of the multilevel perspectives of organizational partners (eg, leaders, managers, staff) and patient partners (eg, family or caregiver, individual).

### Case Example

The multidisciplinary group iteratively developed this approach, and we applied it to design a CDS tool to improve guideline-concordant prescribing of a mineralocorticoid receptor antagonist (MRA) for patients with heart failure and reduced ejection fraction (HFrEF) [49]. Despite strong evidence that MRAs improve mortality and quality of life for patients with HFrEF, suboptimal prescribing remains problematic across health systems [50,51]. We selected this use case because it is widely relevant across health systems and it can be adapted to address other gaps in care.

The MRA CDS tool was designed to be implemented within primary care and cardiology clinics across the UCHealth system. UCHealth is a regional health system that includes 14 hospitals and more than 900 clinics in academic, urban, suburban, and rural settings across Colorado and parts of Wyoming and Nebraska. UCHealth uses one integrated EHR (Epic Systems).

### Ethical Considerations

This study was reviewed and approved by the Institutional Review Board. Patients and clinicians who participated in focus groups provided informed consent and were compensated for their time (patients were compensated $60 and providers were compensated $100). All local, national, regional, and international laws and regulations regarding the protection of personal information, privacy, and human rights were adhered to.

## Results

### Overview

Through a series of iterative applications to the MRA case example and consensus-based discussion with the multidisciplinary group, we developed a 4-step process that can be used to systematically identify and prioritize nudges and that applies to a range of different types of nudges, CDS tools, and diverse clinical issues. These steps are outlined in Textbox 1. The four iterative steps are as follows: (1) engage partners for UCD, (2) develop a shared understanding of the nudge types, (3) determine the overarching CDS format, and (4) brainstorm and prioritize nudges to address each modifiable contextual issue.

Textbox 1. **Stepwise process to applying nudges to clinical decision support (CDS) tools. This approach is guided by the Practical, Robust Implementation and Sustainability Model (PRISM) implementations science framework and leverages the Messenger, Incentives, Norms, Defaults, Salience, Priming, Affect, Commitments, and Ego (MINDSPACE) framework to categorize the various nudge types and the nudge ladder to assess the effectiveness of nudges.**

Step 1: Engage partners for user-centered design

- Assemble a team and clearly define the focus
- Assess partners’ areas of expertise
- Conduct partner engagement to ensure representation of perspectives (equity) and expertise
- Assess contextual facilitators and barriers
- Identify modifiable contextual issues that can be addressed by nudges

Step 2: Develop a shared understanding of nudge types

- Define the nudge types in language that resonates with the team
- Review the anticipated effectiveness of each nudge type

Step 3: Determine the overarching CDS format

- Ensure the CDS format addresses the ultimate intended behavior change
- Consider feasibility of embedded nudges based on the intended behavior change and overarching CDS format

Step 4: Brainstorm and prioritize nudges to address each modifiable contextual issue

- Explore different ways the nudge types can address the modifiable issues
- Promote divergent thinking by having multiple members of the team independently create prototypes
- Develop a plan in place for iterative evaluation of the final product

These steps are iterative and intended to be adapted to align with the local resources and needs of various clinical scenarios and settings. Below, we describe these four steps and illustrate how this approach was applied to the use case of a CDS to improve prescribing of MRAs for HFrEF.

### Step 1. Engage Partners for UCD

Step 1, engage partners for UCD, is a process that involves several sub-steps: assessment of the context, identification of key partners, statement of the problem the CDS will address, and identification of modifiable drivers of the clinical decision.

Aligned with implementation science principles of designing relevant, generalizable, sustainable, and equitable solutions, the first sub-step is using PRISM to assess the context of the local and external setting [52,53]. Key to assessing the context is multilevel partner engagement. PRISM can be used to identify the multilevel partners to engage, inform the input needed from the different partners, and promote equity through representation of all partner perspectives [48]. While CDS are clinician-facing tools, their recommendations should be aligned with the patient’s needs and preferences; therefore, the patient is an important partner perspective to capture. Use of an implementation science framework such as PRISM provides guidance on how to design for equity, sustainability, and scalability (generalizability) from the beginning [53].

Create a clear problem statement or evidence-based gap. This is informed by the contextual assessment and with input from your team. Set a team-based approach with diverse expertise and iterative engagement of multiple types of partners. It is ideal to have expertise in the areas of CDS best practices and technical build, the clinical area of interest, decision science, implementation science, and ethics, but all this expertise is not always available within usual operations. Such expertise may be represented within the “implementation team,” which leads the design activities or through the partner engagement process. Partners engaged should include clinician end users of the CDS and other levels of partners within the local setting who could influence the uptake of the CDS tool (eg, leaders, informatics governance, decision-makers) or who could be influenced by the CDS tool (eg, patients whose care is impacted). The nature of partner engagement can range from formal semi-structured interviews or focus groups to less formal meetings or even emails to meet the goals of the engagement while also fitting within available resources [20].

For the MRA example, the goal was to design a CDS tool to improve evidence-based MRA prescribing for patients with HFrEF in outpatient cardiology and primary care clinics. We convened an implementation team with expertise in the clinical situation, implementation science, clinical informatics, and decision science. We used PRISM with UCD strategies to guide our contextual assessment and partner engagement activities [32]. To understand end user characteristics and perspectives of the CDS solution, we conducted a series of clinician focus groups. Through these focus groups, we assessed contextual factors that influence prescribing, their workflows, and preferences for receiving information within such a CDS solution [54]. Patients with HFrEF were also engaged in focus groups to ensure the CDS recommendations were patient-centered [55]. To promote equity, we used purposeful sampling of providers and patients across different types of settings (eg, academic, rural, and community settings) to ensure representation of diverse perspectives, not just the average. We engaged internal health system operational and informatics leaders and governance groups. This engagement was iterative and occurred via informal one-on-one and standing group meetings to ensure alignment with strategic priorities, gain buy-in, and get approval to deploy the CDS.

Next in the UCD process is understanding modifiable drivers of the clinical decision. Findings from the local context should be iteratively considered and mixed with findings about the context of the external environment (eg, regulations, evidence-based recommendations, reimbursement issues) to understand facilitators and barriers that can influence clinician decision-making, both the initial feasibility and ongoing sustainability of maintaining the CDS tool, and the scalability or generalizability of the tool. Throughout this process, the best practices in CDS design [32], including evidence-based principles of human-computer interaction and UCD methods, provide more granular direction on assessing CDS design decisions that consider issues of socio-technical relationships.

For the MRA example, we quantitatively assessed patterns of prescribing MRAs, including characteristics of patients, to identify additional contextual drivers of prescribing [56]. We then compared our findings to externally published literature to understand generalizability and validate the issues identified. We considered key external issues including national quality benchmarks, evidence-based recommendations for HFrEF management, and best practices in CDS design.

Finally, in Step 1 of the UCD process, modifiable issues are identified that can be addressed by nudges. Not all of what is discovered in Step 1 will be modifiable or amenable to a nudge intervention; therefore, an important part is reviewing the contextual findings to create a clear list of modifiable issues that can be addressed by nudges. Partner input may be needed to determine which issues are modifiable. For example, if the implementation team does not have CDS technical or clinical expertise to consider viability of tangible solutions to contextual issues, these perspectives will need to be engaged. Findings from the contextual assessment in Step 1 that are not amenable to nudge intervention are revisited in later steps to inform the type of nudge selected and its design or form.

In the MRA CDS tool example, the ultimate goal was getting clinicians to prescribe an evidence-based MRA for patients with HFrEF. In Step 1, we identified the following contextual drivers of the decision to prescribe an MRA that were deemed tangible issues for nudges to address within a CDS: (1) remembering to prescribe an MRA during a patient encounter, (2) overcoming clinician misconceptions that high-normal serum potassium and low-normal renal function are barriers to MRA, and (3) time needed to determine what laboratory monitoring is needed to safely monitor an MRA after prescribing. We identified many other contextual barriers and facilitators that were not amenable to a nudge intervention and documented these to consider later. For example, providers felt patients often hesitated to start treatment due to cost or complexity, and while this was considered an important contextual consideration, we felt it was not amenable to a nudge intervention.

### Step 2. Develop a Shared Understanding of the Nudge Types

For Step 2, develop a shared understanding of the nudge types, it is useful to review the nudge types as a team. The focus should be on developing a shared understanding in language that resonates with the team of both the different nudge types and their strength of changing behavior based on the nudge ladder. To facilitate this process, we suggest using MINDSPACE and documenting shared definitions using relatable terminology along with ratings from the nudge ladder for each nudge type. Although MINDSPACE is relatively accessible to broad audiences, the jargon and categorization can still be interpreted in different ways, especially among multidisciplinary teams. We have found this to be an iterative process to come to consensus and, ideally, integrate multidisciplinary team perspectives.

In the MRA CDS tool example, we found that the original definitions of MINDSPACE were not always intuitive and that the differentiation between the categories was not always clear. Because the original MINDSPACE definitions were interpreted differently by different members of our team, this process of developing a shared “mental model” was key to developing a common vocabulary by which we could systematically consider applying the different nudge types. Together, we iteratively worked to create definitions that resonated with the unique perspectives of our multidisciplinary team, that clearly distinguished the different categories of nudge types, and that considered the strength of the nudge. For example, salience was defined as something that draws our attention because it seems novel or relevant, but our team amended this definition to define salience as “anything that draws our attention, not necessarily because it is novel or relevant.” Table 1 provides an example of how we documented our shared understanding of the MINDSPACE nudge types and their strength.

### Step 3. Determine the Overarching CDS Format

The overarching CDS tool itself is a nudge that can include additional embedded nudges. Step 3, determining the overarching CDS format, focuses on determining a format that addresses the ultimate intended behavior change (eg, appropriate prescribing), while the embedded nudges can address additional contextual drivers of the ultimate behavior change (eg, address informational needs). Deciding on the overarching CDS format can be key to guiding later decisions about what embedded nudges are feasible; therefore, Steps 3 and 4 are purposely distinct. For example, with some CDS formats, such as those embedded within a medication order, there may not be space or technical capacity to address additional contextual drivers of the ultimate behavior change. CDS within medication orders often have character limits and do not allow for functions such as hyperlinks, thus providing limited space to address contextual drivers.

Often, the overarching format of the CDS tool is broadly categorized as interruptive (eg, “pop up”) or passive. With passive CDS, either the user needs to seek out the CDS tool or the tool is presented in such a way that the user’s workflow is not interrupted (no hard stop). A decision on whether the overarching CDS tool is an interruptive or passive format is based on the severity and frequency of the targeted behavior to be changed, as well as organizational norms, priority of the issue, and end user preferences [20]. Interruptive CDS is considered a stronger nudge type based on the nudge ladder and is often reserved for higher risk clinical situations. Decisions about the overarching CDS format should be informed by traditional UCD principles and best practices in CDS design [32].

In the MRA example, an interruptive “pop-up” CDS was selected for the overarching format based on the severity of the clinical situation, input from clinicians, and perceived need for space to address additional contextual drivers of prescribing an MRA. Per MINDSPACE and the nudge ladder, this CDS format is a “salient” type, and the strength is “strong.” At our health system, interruptive CDS are reserved for situations in which the clinical severity warrants the interruption or when passive CDS are not feasible to address the need. A strong, salient nudge was deemed appropriate for our local context, and this format provided space to address other drivers of the behavior change.

### Step 4. Brainstorm and Prioritize Nudges to Address Each Modifiable Contextual Issue

Step 4 is to brainstorm and prioritize the nudges. To support divergent and creative thinking, this step is ideally completed independently by multiple members of the team who then share ideas for discussion and prioritization. In this step, each MINDSPACE nudge type defined in Step 3 is iteratively applied or mapped to each modifiable issue identified in Step 2 to define different ways or forms a given nudge type could be designed within the overarching CDS format determined in Step 3. There may be multiple ways a nudge type can be implemented for each modifiable issue, which should be explored. It is also possible there is not a feasible way to apply a given nudge type due to technical or other constraints. When this step is completed by individuals with technical knowledge and understanding of the clinical context, such decisions about feasibility may be more efficient and immediately evident.

In this step, we suggest using visual prototypes or mockups of how the different nudge types could address the different modifiable issues within the overarching CDS format. Mockups can be helpful to efficiently aid understanding and facilitate discussion among the team. In most cases, low-fidelity static prototypes or wireframes are sufficient to illustrate the nudge form and are conducive to rapid iterations.

For the MRA CDS tool example, two clinician informaticists familiar with the clinical situation and technical capability of CDS (KT and SZ) systematically and independently mapped the MINDSPACE nudge types to the modifiable issues identified in Step 2 by creating different forms for each nudge type. They mocked up each nudge form into low-fidelity, static prototypes of the overarching CDS format (interruptive CDS). The prototypes consisted of screenshots of other interruptive CDS that they edited using Microsoft PowerPoint. Based on their knowledge of technical feasibility and clinical situation, they did not identify any practical forms for applying the MINDSPACE nudge types for “Commitments” or “Incentives.” Although the team considered requiring clinicians to provide a free-text reason for deferring prescription that would then be saved to the patient’s EHR record, this was quickly deemed not feasible given technical limitations and thus was not mocked up into a prototype.

Using the mockups, the team then discusses the different forms for each modifiable contextual issue and rates them based on (1) anticipated impact on changing clinician behavior, (2) potential unintended consequences, and (3) technical feasibility to build. In this step, PRISM’s RE-AIM outcomes can prove useful in anticipating the potential impact of a nudge form on pragmatic outcomes of RE-AIM [57,58]. As with any technology, usability and user experience of a given nudge are important determinants to consider when anticipating impact on outcomes, especially those outcomes that are upstream of effectiveness, including clinician adoption and implementation fidelity. Considering the impact of a nudge form based on RE-AIM, including explicit discussion about potential unintended consequences, promotes safe and ethical use of nudges, fosters a culture of “do no harm,” and considers issues of equity and autonomy. RE-AIM can also assist in anticipating the magnitude of impact a nudge form can have on various outcomes including changing clinician behavior and assist in determining if the effort is worth the return [58].

It is important to assess technical feasibility as part of examining the overall effort required. Depending on team composition, ratings of technical feasibility may be reserved for a separate conversation with EHR technical staff (eg, analysts, builders) with this knowledge. A focused discussion regarding the ratio of “benefit to resources required” is important to design practical nudges that consider the impact within resource constraints [33]. For example, if a particular nudge form is anticipated to have low adoption and be moderately effective at changing behavior but will require a high degree of ongoing resource allocation, it may not be prioritized.

For the MRA example, the prototypes guided a discussion between the two clinician informaticists and two other members of the team, a social psychologist and a clinician with expertise in decision science. The team discussed and rated the prototypes based on impact, unintended consequences, and technical feasibility. This discussion and rating process occurred over a series of meetings in which other members of the broader team were consulted as questions arose. For example, the heart failure specialist was consulted when questions arose about the appropriateness of encouraging certain clinical decisions and the operational capacity to include a link for referral to cardiology within the CDS.

Throughout this prioritization process, the nudges with the greatest anticipated value (high impact, minimal or no unintended consequences, low resource burden) are selected and used to iteratively refine CDS mockups that integrate the different combinations of nudge forms within the overarching CDS format in ways that are deemed user-friendly. Principles of UCD and CDS design best practice need to be considered when designing the nudge forms and especially when thinking about how to combine them within the overarching CDS format to optimize human-computer interaction. To promote transparency and generalizability, it is important to document decisions and reasons for decisions. Ultimately, the CDS mockups resulting from this step are then iteratively tested and refined using traditional UCD approaches (eg, simulated scenarios and usability testing) to optimize usability and user experience.

Aligned with the goals of learning health systems, once a minimum viable product is defined, an evaluation plan is critical to ensuring positive impact and to iteratively refine the tool over time. This includes consideration of a study design and pragmatic outcome measurement. PRISM and PRISM’s RE-AIM outcome measures (Reach, Effectiveness, Adoption, Implementation, Maintenance) provide a framework for measuring both implementation and effectiveness outcomes iteratively over time along with the representativeness (equity) of the outcomes and important contextual drivers. More details of how to use PRISM and its RE-AIM outcomes to evaluate CDS tools within learning health systems along with examples can be found in other references [32,59].

For the MRA example, this process led to the development of three low-fidelity static prototypes of the CDS tool that integrated various combinations of the nudge types of salience, messenger, incentives, and defaults in different forms. These three prototypes were iteratively refined by sharing with the broader implementation team and then through a traditional UCD process with potential clinician end users. Examples of the three low-fidelity prototypes and the final CDS tool are available in Multimedia Appendix 1 with illustrative examples of the different nudge types. To evaluate the impact of the MRA CDS tool, we are currently comparing 2 versions of it in a 6-month, mixed methods randomized controlled trial across outpatient cardiology and primary care practices. We are conducting a mixed methods evaluation guided by PRISM and PRISM’s RE-AIM outcomes, including interviews of clinicians and quantitative EHR data.

### Time and Resource Needs

In developing this approach, we initially met frequently, and some meetings were dedicated to trialing different approaches (eg, nominal group technique), but ultimately settled on the approach described here that involved two 1-hour meetings between 2 members of the team (KT and SZ) who rapidly prototyped and prioritized nudge designs, three 1-hour meetings for input from experts in social psychology and decision science (KT, DM, LS, and JM), and periodic asynchronous input via email from other members of the multidisciplinary team (DM, LA, CL, AH, and JM). It is also notable that we did not include a patient perspective in our multidisciplinary group to design the nudges, but we did engage patients early on in Step 1 to ensure the CDS were designed with their preferences in mind.

## Discussion

### Principal Findings and Comparison With Previous Works

We describe a new systematic and pragmatic approach to selection and prioritization of nudges that can be used to comprehensively and systematically consider key issues in designing CDS to optimize feasibility, effectiveness, equity, and sustainability while minimizing potential for unintended consequences. Grounded by implementation science principles and approaches, this 4-step approach involves (1) engaging partners for UCD, (2) developing a shared understanding of the nudge types, (3) determining the overarching CDS format, and (4) brainstorming and prioritizing nudges to address each modifiable contextual issue. The approach prioritizes those applications of nudges with greatest potential to maximize the effectiveness of the intended behavior change, minimize unintended consequences, and align with the variable resource constraints of time and personnel available to design and build CDS tools in ways that are sustainable. We focused on clinician-facing nudges, recognizing they can also be patient-facing [51].

Our findings present one way of systematically and comprehensively selecting nudges for inclusion in CDS tools using an implementation science framework, PRISM [41]. We considered other approaches, including the nominal group technique, but found them to be cumbersome and less practical. The approach we present is intended to be pragmatic, iterative, and rapid within the context of a learning health system and should flex in ways that are relevant and meet the needs and resources or expertise available within a given health system. For example, not all health systems have access to experts in social psychology who have in-depth knowledge of behavioral economics, but this limitation should not preclude them from applying this approach. In other situations, a health system may need to expedite the design and implementation of a CDS solution to address a safety issue [20], and this systematic approach may need to be abbreviated without the luxury of having a series of discussions over time.

Despite growing application of nudges to CDS tools [28-31,60,61], there is a paucity of guidance on how to apply nudges, and recent calls have been made to refine such strategies [62,63]. One framework that others have used to leverage insights from behavioral economics when designing implementation strategies is Easy-Attractive-Social-Timely (EAST) [64,65]. The 4 domains of EAST—easy, attractive, social, and timely—can provide broad guidance when designing nudges for CDS but may not provide the specificity some CDS developers need, nor does EAST provide focused guidance on issues of equity, sustainability, or unintended consequences [64]. In contrast, the approach we describe provides more granular direction on how to select among the various types of nudges by leveraging the MINDSPACE framework [39] while also considering the broad implementation context via its use of the PRISM implementation science framework. Further, our integration of PRISM into this approach provides guidance on how to design nudges in ways that optimize equity and sustainability while minimizing potential unintended consequences of nudges [28-31,41-43,47,48].

When considering the research implications of our findings, this approach addresses an unmet need for guidance on how to systematically apply nudges to CDS tools in ways that allow for adaptation and tailoring to align with the local context, but that is also replicable. This pragmatic and rigorous approach has the potential to optimize the positive impact (eg, effectiveness, equity, sustainability) of CDS tools locally by ensuring relevance, while the systematic process and use of an implementation science framework simultaneously facilitates the scalability of CDS tools and their effect [3]. In using this approach to optimize CDS tools via nudges, it is hoped the well-known knowledge to practice gap can be decreased [66,67].

Aligned with the learning health system goals of being rapid and improving the quintuple aim, we encourage the use of this approach to create minimum viable products that are not anticipated to cause harm [68]. In other words, aim for good enough and not perfect. Technology and health care are rapidly changing, and aiming for perfect is likely an unachievable goal that will only stymie progress. The goal of this systematic approach to nudges is to aim for a CDS solution that is “good enough” while being confident that no harm will result and having a plan for continual improvement over time. This approach provides guidance on how to integrate methods and principles from behavioral economics with implementation science and traditional UCD principles to intentionally design nudges (and other behavioral economics strategies) for CDS that are equitable, effective, sustainable, and generalizable. This approach considers the complexity of how the overarching CDS itself is a nudge which can have layers of embedded nudges that must all work together and align with the dynamically changing context of health care locally and nationally to positively change behavior [69,70].

Table 2 outlines tips for success when using this approach, and Table 3 outlines some considerations for using this approach that surfaced when we developed this approach.

**Table 2.** Recommendations for applying this systematic approach to nudges for CDS^a^ tools.

Iterative step	Keys to success
Engage partners for UCD	Recruit partners within a wide range of disciplines and job roles Conduct an expansive review of contributing factors to the problem, then leverage partner input to delineate which contextual factors are modifiable through CDS Offer multiple formats of partner engagement ranging from participation on the implementation team to occasional emails providing usability feedback Ensure the patient perspective is captured, which may include integrating them within the CDS implementation team
Develop a shared understanding of the nudge types	Select a nudge psychology framework to identify nudge types that is broadly interpretable by a multidisciplinary team Iterate and enhance the shared “mental model” of nudge types using case examples
Determine the overarching CDS format	Do not overlook the format of the CDS tool itself as a potential opportunity to apply nudge psychology, in addition to nudges contained within the tool’s contents
Brainstorm and prioritize nudges to address each modifiable contextual issue	Complete brainstorming steps independently, then engage partners for discussion and prioritization of nudge types and forms Mock up static prototype images of nudge forms to aid understanding and generate feedback Systematically rate each nudge form on anticipated impact, potential unintended consequences, and technical feasibility to build Document the design and decision process for future reuse

a CDS: clinical decision support.

**Table 3.** Frequently asked questions to consider as you apply this approach.

Steps and questions	Answers
Step 1. Engage partners for user-centered design	
What areas of expertise are helpful to engage during this process?	Expertise in CDS^a^ systems, the clinical area of interest, decision science, implementation science, and ethics is helpful whenever feasible. Additionally, consider individuals involved in the entire CDS use cycle, including health system leaders involved in governance decisions, clinician and patient end users, and informatics teams involved in build and maintenance of the tool.
What methods of partner engagement were most effective?	Step 1 emphasizes the importance of diverse user-centered and multi-level perspectives, but this must be balanced with resource and scalability constraints. We found success in varying the formality and format of engagement activities depending on the goals of the engagement (eg, semi-structured interviews and focus groups for brainstorming, one-on-one meetings for testing technical build options). This facilitated increased representation without compromising efficiency.
Step 2. Develop a shared understanding of the nudge types	
Did the team encounter any challenges with applying a nudge framework to technical build?	Not all nudge types are easily translatable into a CDS solution. However, key to this approach is the brainstorming process where the team explored many different ways to implement each nudge type, prior to rating the solutions based on technical feasibility and impact. This encouraged “out-of-the-box” problem-solving that did not rely on local standards or vendor capabilities.
Step 3. Determine the overarching CDS format	
Are there differences in the strategy for determining the overarching nudge type versus embedded nudges?	While the nudge ladder can inform embedded nudges as well, we found it particularly helpful for determining the nudge type of the overarching CDS format as it correlates well with existing CDS best practices such as aligning intrusiveness and frequency with perceived risk. Additionally, the sustainability and generalizability of the overarching CDS format should be considered.
Step 4. Brainstorm and prioritize nudges to address each modifiable contextual issue	
What are some examples of potential unintended consequences?	We considered designing a nudge type of norm to compare each clinician’s prescribing rate to their peers but ultimately decided that this could lead to complacency if a given clinician was performing better than their peers.

a CDS: clinical decision support.

The approach we present to selecting nudges should be used as a guide and adapted as needed. When adapted, we encourage documenting and reporting how the process was adapted, to promote rigor and replicability. Those seeking to replicate this approach for their health system may also choose to use a different implementation science framework that is more familiar to them or that fits their situation better [71,72]. There are also other frameworks to categorize nudge types that could be used [25].

### Strengths and Limitations

Limitations of this approach include the costs of personnel time incurred from multiple meetings; these are important to consider and balance when deciding how to apply this approach. Although our use of a multidisciplinary team-based approach is a strength, not all health systems will have access to the same types of expertise. In this paper, we describe how one health system applied the approach based on their available resources and expertise, which may not be generalizable for all health systems. However, this approach is intended to be pragmatic, and we encourage health systems to adapt it to fit within the available resources and expertise. Further, although this team-based approach may rely on consensus for decision-making, decisions are grounded in evidence-based principles of human-computer interaction and guided by principles of UCD and conceptual frameworks, namely MINDSPACE and PRISM from implementation science.

### Conclusions

By using a systematic approach to selecting nudges and documenting and reporting reasons for decisions, the findings can be adapted and generalized to other health settings and clinical situations, advancing the goals of learning health systems to generate evidence that is both internally and externally valid [3]. Grounded in implementation science, this approach has the potential to improve the effectiveness, equity, and sustainability of CDS tools while minimizing the potential for unintended consequences. We hope that lessons learned using this and similar approaches will be used to make these approaches more accessible and useful to broad audiences to optimize the effectiveness of CDS and ultimately expedite the translation of evidence into practice. Future research is also needed to evaluate the impact of using this approach on CDS effectiveness.

## Supplementary material

10.2196/73189 Multimedia Appendix 1 Prototypes of a Clinical Decision Support (CDS) Tool to Improve Guideline-Concordant Prescribing of Mineralocorticoid Receptor Antagonists (MRA) for Patients with Heart Failure.

## Abbreviations

CDS: clinical decision support
EAST: Easy-Attractive-Social-Timely
EHR: electronic health record
HFrEF: heart failure with reduced ejection fraction
MINDSPACE: Messenger, Incentives, Norms, Defaults, Salience, Priming, Affect, Commitments, and Ego
MRA: mineralocorticoid receptor antagonist
PRISM: Practical, Robust Implementation and Sustainability Model
RE-AIM: Reach, Effectiveness, Adoption, Implementation, Maintenance
UCD: user-centered design
