# Supplementary material for: Application of Nudges to Design Clinical Decision Support Tools: Systematic Approach Guided by Implementation Science
Source: J Med Internet Res. 2025 Sep 25;27:e73189. doi: 10.2196/73189 (PMC12463335; doi:10.2196/73189)
Supplement: Multimedia Appendix 1 [file jmir-v27-e73189-s001.docx]

**Prototypes of a Clinical Decision Support (CDS) Tool to Improve Guideline-Concordant Prescribing of Mineralocorticoid Receptor Antagonists (MRA) for Patients with Heart Failure.^*^**

Figure S1: Prototype Containing Referral Order to Heart Failure Specialist as a Recommended Action

**
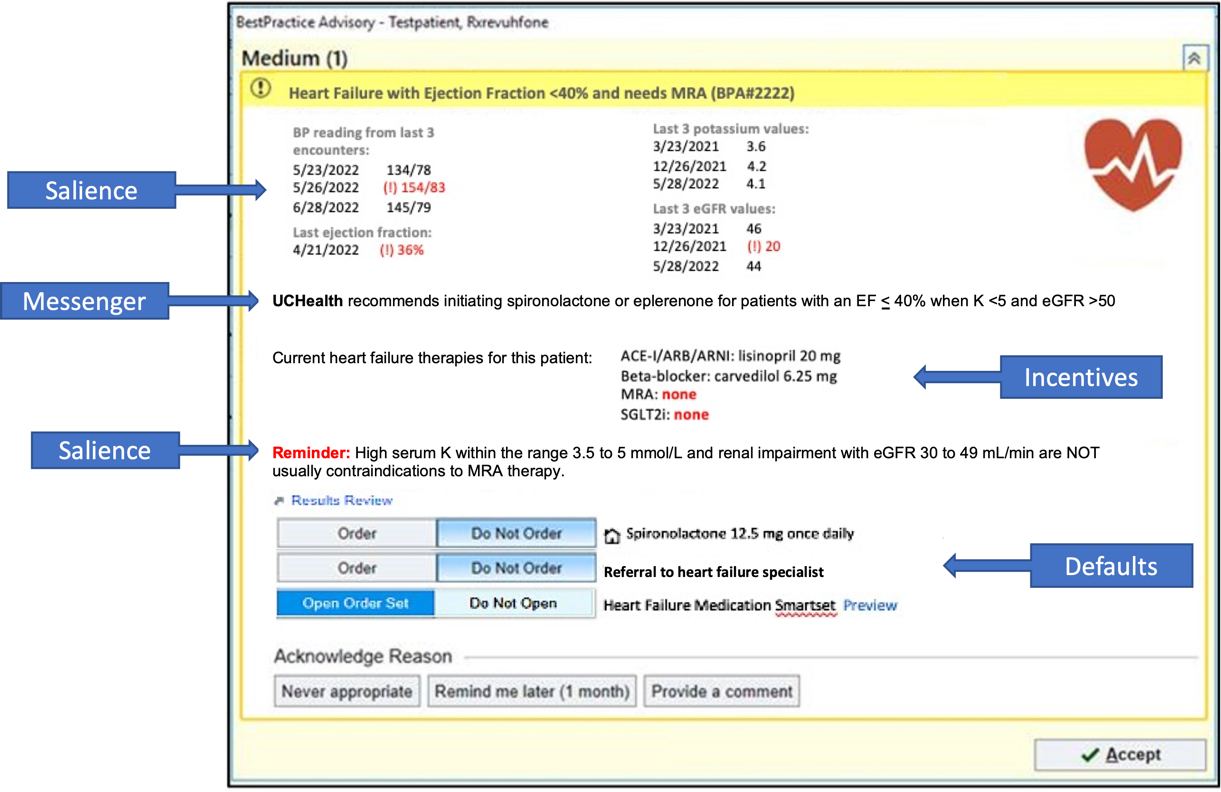
**

© 2025 Epic Systems Corporation

Figure S2: Prototype Containing Lab Orders as a Recommended Action


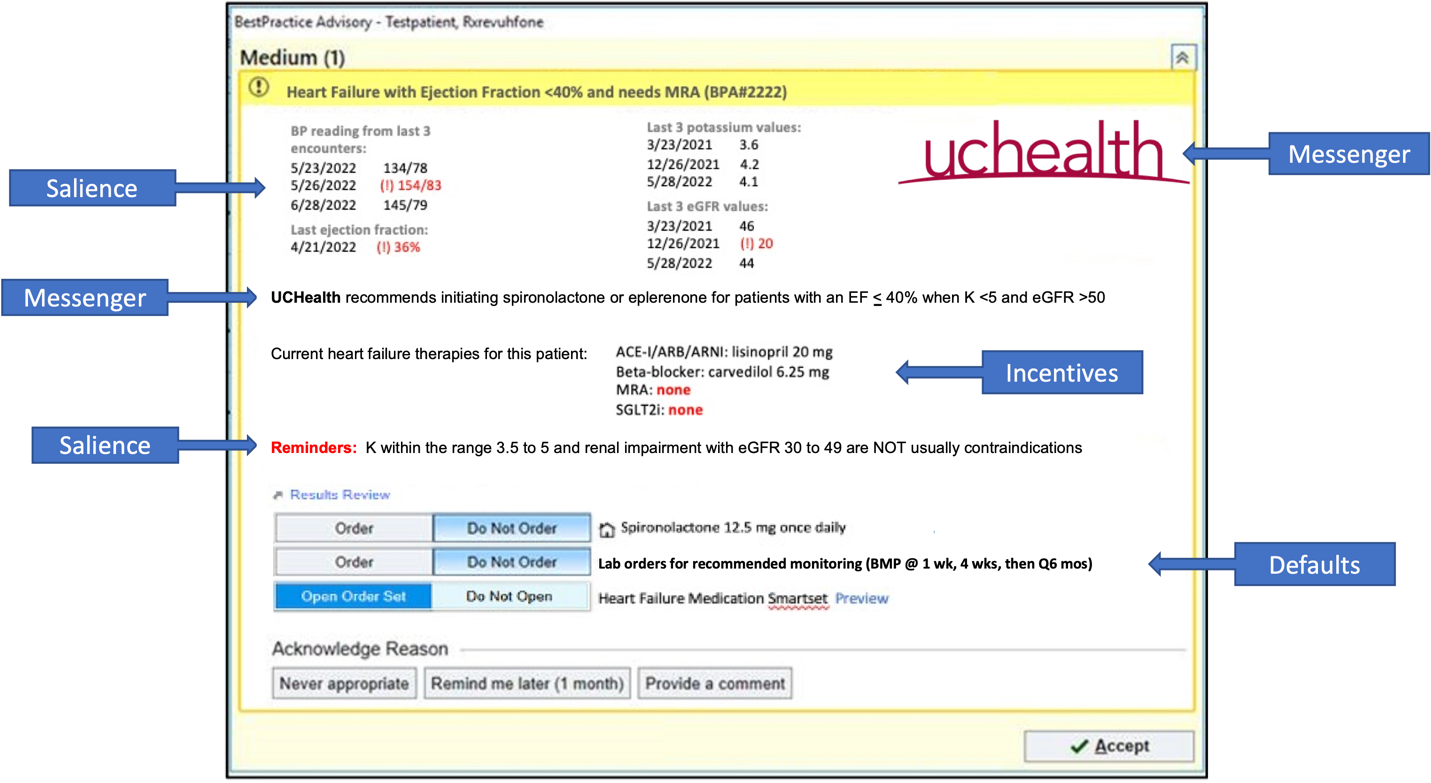


© 2025 Epic Systems Corporation

Figure S3: Prototype Containing Multiple MRA Order Options as Recommended Actions


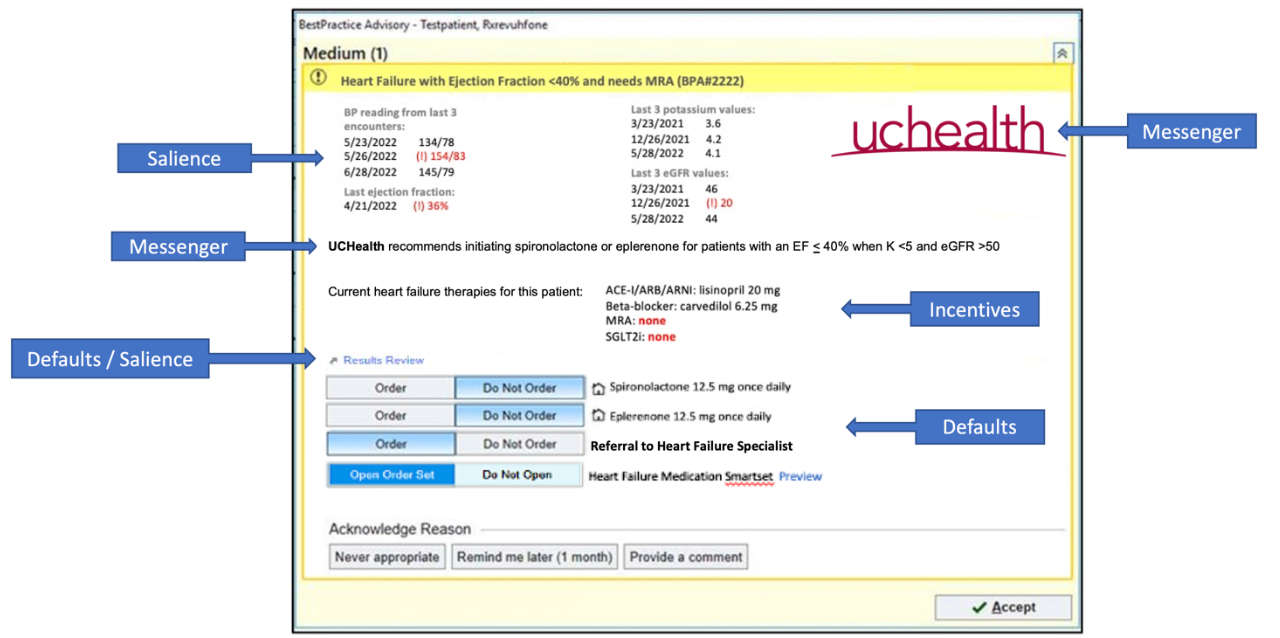


© 2025 Epic Systems Corporation

Figure S4: Final Iteration of the CDS Tool
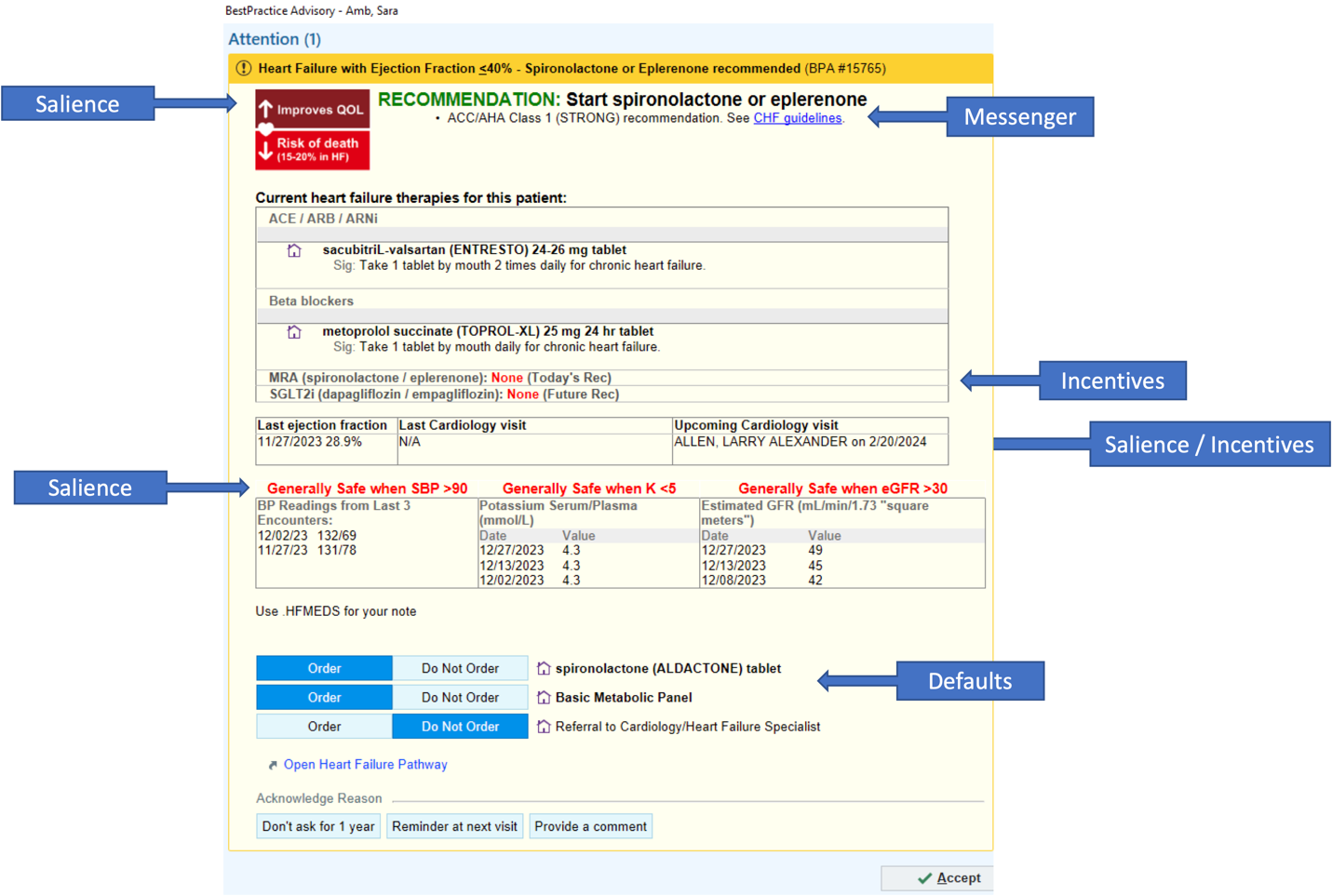


© 2025 Epic Systems Corporation

*Low-fidelity static prototypes (Figures S1-S3) were developed using Microsoft PowerPoint as a wireframing tool, rather than directly within the electronic health record. This enabled efficient concept design and iteration.

This is a Multimedia Appendix to a full manuscript published in the J Med Internet Res. For full copyright and citation information see http://dx.doi.org/10.2196/jmir.xxxx.
